# Strength Training Improves Fatigue Resistance and Self-Rated Health in Workers with Chronic Pain: A Randomized Controlled Trial

**DOI:** 10.1155/2016/4137918

**Published:** 2016-10-17

**Authors:** Emil Sundstrup, Markus Due Jakobsen, Mikkel Brandt, Kenneth Jay, Per Aagaard, Lars Louis Andersen

**Affiliations:** ^1^National Research Centre for the Working Environment, Copenhagen, Denmark; ^2^Department of Sports Science and Clinical Biomechanics, SDU Muscle Research Cluster, University of Southern Denmark, Odense, Denmark; ^3^Physical Activity and Human Performance Group, SMI, Department of Health Science and Technology, Aalborg University, Aalborg, Denmark; ^4^The Carrick Institute, Clinical Neuroscience and Rehabilitation, Cape Canaveral, FL 32920, USA

## Abstract

Chronic musculoskeletal pain is widespread in the working population and leads to muscular fatigue, reduced work capacity, and fear of movement. While ergonomic intervention is the traditional approach to the problem, physical exercise may be an alternative strategy. This secondary analysis of a randomized controlled trial investigates the effect of strength training on muscular fatigue resistance and self-rated health among workers with chronic pain. Sixty-six slaughterhouse workers with chronic upper limb pain and work disability were randomly allocated to 10 weeks of strength training or usual care ergonomic training (control). At baseline and follow-up, participants performed a handgrip muscular fatigue test (time above 50% of maximal voluntary contraction force) with simultaneous recording of electromyography. Additionally, participants replied to a questionnaire regarding self-rated health and pain. Time to fatigue, muscle strength, hand/wrist pain, and self-rated health improved significantly more following strength training than usual care (all *P* < 0.05). Time to fatigue increased by 97% following strength training and this change was correlated to the reduction in fear avoidance (Spearman's rho = −0.40; *P* = 0.01). In conclusion, specific strength training improves muscular fatigue resistance and self-rated health and reduces pain of the hand/wrist in manual workers with chronic upper limb pain. This trial is registered with ClinicalTrials.gov NCT01671267.

## 1. Introduction

The prevalence of work-related musculoskeletal disorders in the hand, wrist, and upper extremity is high in occupations involving highly repetitive movements. The etiology of upper limb work-related musculoskeletal pain is multifaceted encompassing both heavy manual labor, high pace, lack of sufficient recovery, awkward postures, and fatigue [1, 2]. In addition to the more generic risk factors associated with work-related pain, slaughtering and meat processing operations involve several individual determinants associated with upper limb pain: high movement velocity and accelerations, high external force demands, repetitive movements, and prolonged activity with little variation [1, 3]. The occurrence of musculoskeletal pain in the shoulder, arm, and hand regions is particularly prevalent among slaughterhouse workers probably due to the high degree of repetitive and forceful upper limb movements exerted during work [4–6]. In a recent cross-sectional study among more than 600 Danish slaughterhouse workers and with a response rate of 92%, the prevalence of pain in the shoulder, elbow, and hand/wrist was 60%, 40%, and 52%, respectively, while 38% of the workers reported work disability due to upper limb pain [7]. The present study represents an interventional model to reduce work-related upper limb pain and its consequences.

Fatigue is a frequently reported complaint among the general working population and can be viewed as an imbalance between demands of the job and the workers ability or capacity to perform the job [8]. Long-term fatigue increases the risk of work disability and long-term sick leave [9, 10]. Thus, evaluation of neuromuscular fatigue development has gained a lot of attention the previous years. Nevertheless, only few previous studies have tested interventions to counteract fatigue development among persons with chronic diseases, and studies on the potential effects of different rehabilitation strategies on muscle fatigue development are needed. Muscular fatigue expresses acute impairment in motor performance encompassing both increased perception of task difficulty and impaired ability to maintain the desired level of force production [11]. Muscular fatigue is associated with decreased force output, decreased movement velocity, decreased range of motion, motor variability, increased EMG amplitude, and a shift towards lower frequencies in the EMG power spectrum [12, 13].

Musculoskeletal pain aggravates development of fatigue [14, 15]. Hansson et al. [14] reported reduced shoulder endurance time as a function of neck/shoulder pain among women with repetitive work compared with asymptomatic controls. In that study, the most pronounced sign of neuromuscular fatigue was increased trapezius EMG activity and a decrease in median power frequency in the deltoid muscle. As chronic pain is a multifactorial experience, it has additionally been suggested that pain-related fear, such as fear avoidance, may limit efferent neural motor drive [16] and thus lead to impaired physical performance among individuals with pain [17]. It can be speculated that the observed impairments in mechanical muscle function in workers with chronic pain would lead to increased job strain and directly contribute to impaired endurance. Interventions to reduce pain and increase neuromuscular function, respectively, might have the potential to postpone muscular fatigue development and thus improve work capacity and elicit general health benefits among workers with chronic pain.

Within the recent decade physical exercise has been increasingly used in the prevention and treatment of chronic diseases [18]. Previous occupational studies have shown promising effects of reducing pain by increasing physical capacity through strength training at the workplace [19, 20]. However, only few studies have investigated whether increased muscle strength and reduced musculoskeletal pain can be transferred to improved work capacity evidenced by increased muscular endurance and overall enhanced fatigue resistance. Andersen et al. [15] demonstrated that 10 weeks of strength training effectively improved strength-endurance capacity of female office workers with diagnosed trapezius myalgia. However, results from office workers may not at all be generalizable to slaughterhouse workers with chronic pain.

The aim of the present study was to investigate the effect of specific strength training and usual care ergonomic training on skeletal muscle fatigue resistance (i.e., the ability to resist fatigue) and pain in the hand/wrist along with pain related beliefs and self-rated health among slaughterhouse workers with upper limb chronic pain and work disability. It was hypothesized that strength training would be superior to usual care ergonomic training with regard to improved fatigue resistance of chronically painful muscles.

## 2. Materials and Methods

### 2.1. Study Design

The data reported in the present article represents a secondary analysis of a randomized controlled trial focusing on rehabilitation of chronic upper limb pain. The study protocols along with primary and secondary outcomes have been reported elsewhere [20, 21]. The study was approved by the Danish National Ethics Committee on Biomedical Research (Ethical committee of Frederiksberg and Copenhagen; H-3-2010-062) and was registered in ClinicalTrials.gov (NCT01671267) prior to enrolment of the first participant. Experimental conditions conformed to the Declaration of Helsinki and all participants gave their informed written consent to participate in the study.

We used a single-blind randomized controlled study design with allocation concealment in a two-armed parallel group format among 66 slaughterhouse workers with chronic pain and work disability. Using a random numbers table generated in the SAS statistical software, participants were randomly allocated to receive either strength training or ergonomic training (usual care control group) for 10 weeks at the slaughterhouse. The person who performed the randomization e-mailed the information about group allocation to a representative at the slaughterhouses, who provided the information to the enrolled participants. At baseline and follow-up, participants performed various muscular fatigue and strength tests, underwent a clinical examination of the shoulder, arm, and hand, and performed a questionnaire survey concerning perceived pain, pain related beliefs, and self-rated health. All interventional activities and data collection took place at the two enrolled slaughterhouses in Denmark, Europe. Baseline characteristics of all participants are listed in Table 1.

### 2.2. Inclusion of Participants

Inclusion criteria for participation in the study were as follows: (1) working at a slaughterhouse for a minimum of 30 hours/week, (2) pain intensity in the shoulder, elbow/forearm, or hand/wrist ≥3 on a 0–10 VAS during both the last week and last 3 months, (3) pain lasting ≥3 months, (4) frequency of pain of ≥3 days/week during the last week, (5) work disability due to chronic pain in the shoulder, elbow/forearm, or hand/wrist, and (6) no participation in strength training and no receiving of ergonomic instructions during the last year. All six criteria had to be met. Exclusion criteria were hypertension (systolic blood pressure >160, diastolic blood pressure >100), a medical history of cardiovascular diseases, carpal tunnel syndrome, recent traumatic injury of the neck, shoulder, arm, or hand regions, or pregnancy.

**Figure 1:**
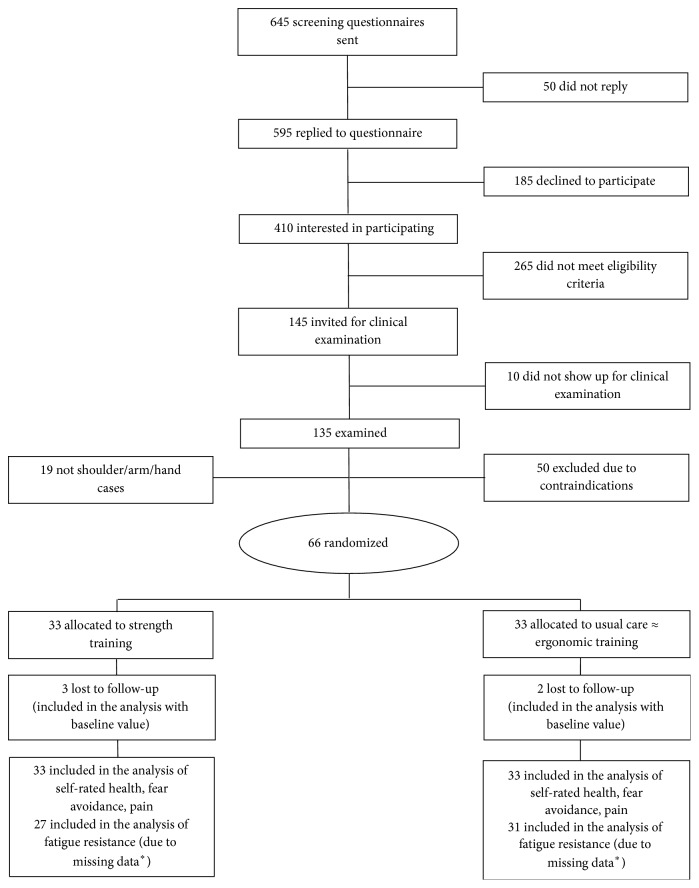
Participant flow. ^*∗*^Missing data (i.e., participants with no data on fatigue test at baseline and follow-up) was present from 6 and 2 participants in the strength training and in the usual care ergonomic group, respectively. Therefore, 27 and 31 participants were included in the analyses of the fatigue test from the strength training group and usual care ergonomic group, respectively.

A total of 135 participants were included for the baseline clinical examination, of which 69 were excluded due to the following contraindications: 19 showed symptoms of carpal tunnel syndrome, 4 had blood pressure above 160/100 mmHg, 1 had a serious cardiovascular disease, and 19 did not meet the pain inclusion criteria. Furthermore, 26 were excluded because they did not speak or understand Danish sufficiently to respond to the questionnaire. The overall flow of participant enrolment is depicted in Figure 1 and has previously been described in detail [20].

### 2.3. Blinding Procedures

All examiners were blinded to group allocation during the physical test procedures and questionnaire assessments performed at follow-up. Additionally, participants were carefully instructed not to reveal their particular group allocation. The design of the study makes blinding of participants and instructors impossible (i.e., strength training and ergonomics instructors); however, at baseline, the participants had similar outcome expectations to the two interventions concerning the effectiveness on chronic pain [20]. All outcome assessors were blinded to group allocation.

### 2.4. Sample Size

Sample size was calculated on the primary outcome parameter (pain intensity) as reported elsewhere (31). In brief, our analysis indicated that 27 participants in each group were needed for testing the null hypothesis of equality of treatment at an alpha level of 5%, a statistical power of 95%, a minimally relevant difference in pain intensity of 1.5, and assuming an overall SD of 1.5 (0–10 scale). With an estimated participant dropout/loss at follow-up of 10%, the minimum number of participants in each group at baseline was found to be 30.

### 2.5. Intervention Procedures

Participants were randomized to receive specific strength training (*n* = 33) conducted in designated facilities at the workplace using an exercise program designed to target muscles in the shoulder, arm, and hand 3 times a week for 10 minutes throughout the 10-week intervention period. The training program consisted of the following resistance exercises: shoulder rotation in two planes with elastic tubing, ulnar and radial deviation of the wrist using sledgehammers, eccentric training of the wrist extensors using a flexbar (Tyler twist), wrist flexion and extension by the use of a wrist roller, flexion of the hand using a hand gripper, and extension of the hand using hand bands. Skilled instructors supervised all training sessions and coached in using correct exercise techniques and, when needed, performing individual exercise adjustments. Training intensity was progressively increased from using 20 repetition maximum (20 RM) loads during the first weeks of training to approaching 8 RM loading intensities during the later training phase (all sets performed to contraction failure) according to the principle of periodization and progressive overload [22]. Three to four different exercises (of the 8 available exercises) were performed for 3 sets during each training session in an alternating manner. During each training session, a new exercise-set was starting every minute, but due to the alternating manner of the program, there was approximately 3 minutes between each set of the same exercise. In addition, instructors were to immediately report any adverse events of the training (e.g., dropping the training equipment over the foot or acute strain of the shoulder, arm, or hand) to the researchers. Portable exercise equipment, for home training in case of absence from work (e.g., vacation), was additionally administered to the participants.

Participants randomized to the usual care control group (*n* = 33) received ergonomic training with particular focus on job specific hands-on training through appropriate guidance and training in how to handle the individual work stations. The ergonomic guidance and training program took place during the initial weeks of the 10-week intervention period which corresponds to the standard worksite ergonomic prescription. The intervention was implemented by experienced health and safety representatives employed by the slaughterhouses.

### 2.6. Outcome Measurements

At baseline (August-September 2012) and follow-up (December 2012-January 2013) participants performed muscular strength and fatigue tests and replied to a questionnaire concerning pain intensity, fear avoidance, and self-rated health. Details of the testing procedure are described below.

#### 2.6.1. Pain Intensity

Changes in hand/wrist pain intensity from baseline to 10-week follow-up were rated subjectively using the 0–10 modified VAS scale, where 0 indicates “no pain at all” and 10 indicates “worst pain imaginable” [23]. Participants were instructed to rate experienced pain intensity during the last 7 days of the study period, and the hand/wrist region was explicitly defined to all study participants by drawings from the Nordic questionnaire [24].

#### 2.6.2. Maximal Voluntary Isometric Contraction (MVC)

Participants performed two isometric handgrip MVCs, interspersed by a 30 sec rest period, with their dominant hand using a hand held dynamometer connected to a personal computer (Biometrics Ltd., Ladysmith, VA, USA) [25]. Participants were positioned on a chair in an upright position and with the elbow of the dominant side flexed at a 90°. The dynamometer was held in a neutral wrist position while the nondominant arm was hanging straight down the nondominant side of the body. During the MVCs, participants were instructed to press as fast and hard as possible for approximately 5 sec [16]. Strong verbal encouragement and online feedback of the force exerted were provided to the subject during all trials. The trial with the highest peak force was used for the subsequent analysis and for normalization during the fatigue test. Additionally, an isometric hand extension MVC was performed in a custom-built dynamometer to serve as normalization for the EMG measurement of the extensor carpi radialis brevis muscle (described in detail below) [20]. We additionally performed a reliability study (*n* = 31) showing excellent between-day reliability of the MVC tests used in the present study (ICC > 0.94 and measurement error < 8%).

#### 2.6.3. Fatigue Test

**Figure 2:**
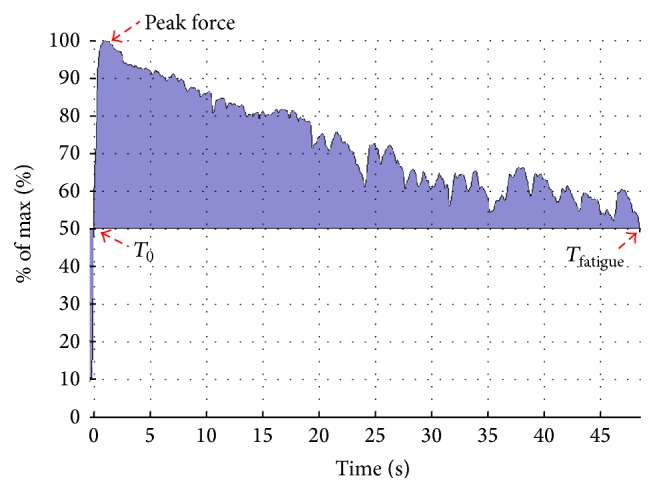
A representative illustration of the force output during the handgrip fatigue test in a strength trained person at follow-up. Individuals were to press as hard as possible throughout the entire fatigue test, and when the force output decreased to below 50% of MVC the test was finished. Peak force (normalized to MVC), start time (*T*_o_), and time at fatigue (*T*_fatigue_) are illustrated on the figure.

Participants performed a muscle fatigue test for the lower arm using the handgrip dynamometer described above. Participants were instructed to produce as much handgrip force as possible for as long as possible. The test was abolished when the force output had declined to below 50% of handgrip peak force (Figure 2). Online feedback and strong verbal encouragement were given during all fatigue tests performed. Raw EMG and MVC force signals were synchronously sampled at 1000 Hz and subsequently low pass filtered (15 Hz cut-off frequency, 4th-order zero-lag Butterworth filter) using a custom-made MATLAB program (MathWorks). Time to fatigue (time to <50% peak force) and total force-impulse (area under the force-time curve, Newtons by seconds) were determined from the fatigue test. Figure 2 illustrates representative force output tracings.

#### 2.6.4. EMG Processing and Data Analysis

To assess the magnitude of neuromuscular activity electromyography (EMG) signals were recorded from the extensor carpi radialis brevis and flexor carpi radialis brevis during the MVC trials and the fatigue tests, respectively. Bipolar surface electrodes (Blue Sensor, Ambu A/S, Ballerup, Denmark) were placed on the lower arm (dominant side) with an interelectrode distance of 2 cm. Before affixing the electrodes, the skin of the respective area was shaved and prepared with scrubbing gel (Acqua gel, Meditec, Parma, Italy) to effectively lower the impedance. Electrode placements followed SENIAM recommendations (http://www.seniam.org/). The electrodes were connected through thin shielded cables to a data-logger (Nexus10, Mind Media, Netherlands) that was placed in a flexible belt to ensure mobility during the testing procedure. EMG activity was sampled at 1,024 Hz. To ensure quality of the EMG signals, all recorded signals were visually inspected. Data filtering and data analysis were performed using custom-made MATLAB programs (MathWorks).

During later offline analysis, all raw EMG signals obtained during the MVC trial and the fatigue tests were digitally high-pass filtered using a Butterworth 4th-order high-pass filter (10 Hz cut-off frequency). For each individual muscle (i.e., flexor carpi radialis brevis and extensor carpi radialis brevis), a moving root-mean-square (RMS; 500-ms time constant) filtering routine subsequently was used to smooth the EMG signal following which peak EMG amplitude was identified [26].

In the fatigue test, EMG peak amplitude (absolute values and normalized to MVC) and mean EMG amplitude were determined as the peak value and average value of the filtered EMG-time curves, respectively. Additionally, using Fast-Fourier transformation (FFT) the spectral density of the raw high-pass filtered EMG signal was evaluated by calculating median power frequency (MPF).

#### 2.6.5. Self-Rated Health

Self-rated health was evaluated with the single global health-rating item from the Medical Outcomes Survey 36 item short form (SF-36) questionnaire [27]. Participants responded to the question “how do you rate your overall current health?” on a 5-point Likert scale ranging from 1 (excellent) to 5 (poor).

#### 2.6.6. Fear Avoidance

Fear avoidance was evaluated using a tailor-made single-item question before and after the intervention period. Participants responded to the question: “fast and forceful arm movement exacerbates pain in my shoulder, arm, or hand?” on a 4-point Likert scale with the response options: 1 (not at all), 2 (a little), 3 (some), and 4 (a lot) [28]. For further statistical analysis, the 1–4 scale was used to identify the level of fear avoidance, meaning that values close to 1 represent no fear avoidance, and levels close to 4 represent a high level of fear avoidance. We included this question because slaughterhouse work tasks frequently encompass fast and forceful upper limb movement. However, this question did not ask specifically about fear avoidance at work, but during fast and forceful movements in general. Thus, the results based on this question may only be relevant during activities that are fast and forceful.

### 2.7. Statistical Analysis

All statistical analyses were performed using SAS statistical software for Windows (SAS Institute, Cary, NC). All outcome parameters were analyzed according to the intention-to-treat principle by use of mixed linear model analysis using a repeated measures 2 × 2 mixed factorial design (Proc Mixed), with* time*,* group*, and* time by group* as independent categorical variables (fixed factors). Each participant was entered as a random effect and analyses were adjusted for gender, workplace location, age, and dependent variable at baseline. The Proc Mixed procedure inherently accounts for missing values. Correlation analyses (Spearman's rho) were performed to evaluate potential associations between changes in fear avoidance, pain, time to fatigue, muscle strength, and muscle activation following the period of intervention. Results are reported as between-group least square mean differences and 95% confidence intervals from baseline to follow-up unless otherwise stated. An alpha level of 0.05 was used to denote statistical significance.

## 3. Results

Table 1 shows baseline characteristics of all study participants. At baseline, age was slightly higher in the strength training group compared with the ergonomic usual care group (*P* = 0.05). This was controlled for in the statistical analysis by including age as a covariate. No significant group differences were observed for any other outcome variables.

### 3.1. Adherence

Adherence to the ergonomic training program was 97%, as one participant refrained from receiving ergonomic training. The strength training group performed on average 2.4 of the three intended training sessions per week, corresponding to a training adherence of 81%.

### 3.2. Dropouts, Missing Data, and Adverse Events

Five participants did not present for the follow-up examination, which comprised three participants in the strength training group and two in the usual care ergonomic group (Figure 1). In the ergonomic training group, one participant dropped out due to job transfer and one participant dropped out due to illness unrelated to the ergonomic training program. In the strength training group, one participant dropped out due to job transfer, one participant dropped out due to illness unrelated to the training program, and one participant dropped out due to training having no subjective effect on upper limb pain intensity. No adverse events of the strength training program were reported by the training instructors.

Missing data (i.e., no data on fatigue test at baseline and follow-up) was present from 6 and 2 participants in the strength training and in the usual care ergonomic group, respectively (Figure 1), whereas, missing data on the handgrip test was present in 4 and 2 participants, respectively. Specifically, in the strength training group, six and one participants did not perform the handgrip MVC test at baseline and follow-up, respectively. In the ergonomic group, three participants did not perform the baseline MVC test. Ten participants in the strength training group and eleven participants in the ergonomic group did not perform the fatigue test at baseline, whereas this was the case for five and three participants at follow-up, respectively. Missing EMG data from the MVC and fatigue tests were present in 0–7% of the participants.

### 3.3. Maximal Muscle Strength

A group-by-time interaction was observed for handgrip maximal isometric muscle strength (*P* < 0.0001). Compared with the ergonomic usual care group, handgrip strength increased to a greater extent in the strength training group (Table 2). Within-group differences were observed as well, with the strength training group increasing MVC strength by 11% (*P* < 0.01) whereas ergonomic usual care subjects demonstrated a decrease in MVC muscle strength by 16% (*P* < 0.01).

### 3.4. Fatigue Test

A group-by-time interaction was observed for time to fatigue (*P* < 0.001). Compared with the ergonomic control group, time to fatigue improved by 23.5 seconds in the strength training group corresponding to a within-group improvement of 97% (*P* < 0.0001, Table 2). No within-group changes were observed in the ergonomic group (Table 2).

A comparable group-by-time interaction was observed for contractile impulse produced during the fatigue test (area covered by the force-time curve) (*P* < 0.001). Postintervention impulse increased by 96% in the strength training group (*P* < 0.0001) whereas no within-group change was observed in the ergonomic group (Table 2).

### 3.5. Neuromuscular Activity

Group-by-time interactions were also present for absolute extensor peak EMG amplitude and absolute extensor mean EMG during the fatigue test (*P* < 0.01 and *P* < 0.05, resp.). Extensor muscle peak EMG and mean EMG improved by 24% and 18%, respectively, following the period of strength training, whereas no significant changes were observed in response to ergonomic usual care (Table 2). No group-by-time interaction was observed for any of the remainder EMG variables obtained during the fatigue test (i.e., flexor peak EMG, flexor mean EMG, and median power frequency; Table 2).

No group-by-time interactions were observed for extensor and flexor EMG signal parameters obtained during the handgrip MVC (*P* = 0.18 and *P* = 0.9, resp., Table 2).

### 3.6. Pain Intensity

**Figure 3:**
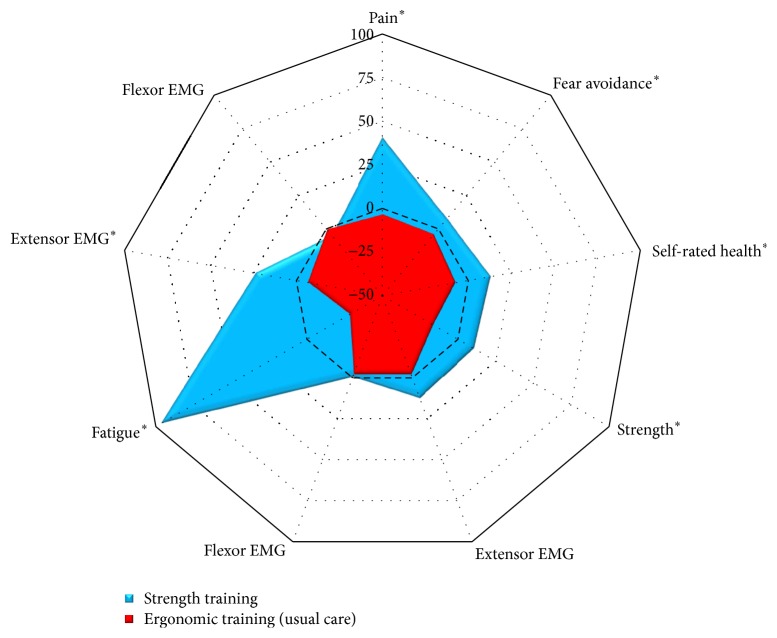
Radar-chart summarizing the interventional changes (% change) for the main variables following strength training (blue covered area) and ergonomic usual care (red covered area). The black broken line represents no change. Variables arranged clockwise: (1) pain intensity of the hand/wrist, (2) fear avoidance, (3) self-rated health, (4) handgrip strength, (5) extensor EMG during handgrip MVC, (6) flexor EMG during handgrip MVC, (7) time to fatigue, (8) extensor EMG during fatigue test, (9) flexor EMG during fatigue test. ^*∗*^ *P* < 0.05.

A group-by-time interaction for pain intensity in the hand/wrist was observed (*P* < 0.01). Compared with the ergonomic training group, pain intensity of the hand/wrist decreased by 1.6 (−2.4 to −0.8) in the strength training group, corresponding to a within-group pain reduction of 41% (Figure 3).

### 3.7. Self-Rated Health

A group-by-time interaction was observed for self-rated health (*P* = 0.026). Compared with the ergonomic group, self-rated health improved to a greater extent with strength training (−0.42 [−0.9 to −0.1]). Self-rated health was improved following strength training (−0.4 [−0.7 to −0.1]), whereas no significant within-group change was observed following ergonomic training (0.2 [−0.2 to 0.5]).

## 4. Discussion

The main finding in the present study was that 10 weeks of specific strength training at the workplace led to increased muscle strength, improved muscular fatigue resistance, and diminished pain rating in the hand/wrist region, accompanied by improved self-rated health in manual workers with chronic upper limb pain and work disability.

In the present group of workers with chronic upper limb pain we have previously demonstrated reduced pain intensity and diminished work disability in response to 10 weeks of strength training compared with usual care ergonomic training [20]. The present study elaborates on these findings by demonstrating that strength training improves muscular fatigue resistance and thus leads to elevated work capacity and thereby increased reserve capacity of chronically painful hand/wrist muscles in slaughterhouse workers with chronic upper limb pain. This is likely to have significant functional impact on high-load upper limb slaughterhouse work by enhancing the ability to sustain a high force production and thus potentially prevent or delay development of acute and accumulated fatigue during the working day. In resemblance with the observed improvements in hand/wrist pain, strength, and self-rated health, strength training appears to represent a potent modality of workplace-based exercise intervention to reduce potential imbalances between individual capacity and work demands, consequently providing a basis for improvements in employee work ability and overall health.

Our data revealed that 10 weeks of strength training was more effective than usual care ergonomic training in improving relative muscular endurance during the handgrip fatigue test. Specifically, strength training led to a 97% increase in time to fatigue (i.e., 50% of peak force) from baseline to follow-up. The strength training program consisted of specific resistance exercises that targeted the painful muscles also evaluated in the fatigue test (e.g., the handgrip and wrist-roller exercises). Notably, all sets were performed until contraction failure. This training design may likely explain the substantial improvements observed in relative muscular endurance following the period of interventional strength training. The improved fatigue resistance could also have been influenced by the observed reduction in pain intensity, which likely would have led to a higher force exertion for a longer period of time. Several factors could potentially have contributed to the reduction in pain observed following the strength training intervention. The reduction could possibly be ascribed to the direct effect of the implemented training program on the painful muscles. For instance, previous studies have shown that exercise can reduce systemic inflammation [29–31]. Specifically, contracting muscles secrete cytokines with anti-inflammatory properties [29, 32, 33], suggesting that the contractions performed during the training sessions in the present study could have influenced the results on pain intensity. Importantly, the training instructors were to report any adverse events to the researchers, for example, dropping the training equipment over the foot, or acute strain of the shoulder, arm, or hand. No such adverse events were observed with strength training intervention, and the specific program performed therefore seems safe to implement among slaughterhouse workers with chronic pain.

The positive change in fatigue resistance was accompanied by a 24% increase in hand extensor peak EMG amplitude during the fatigue test, thus demonstrating increased neuromuscular activity during a fatiguing motor task in the painful muscle following strength training. However, no corresponding change in neuromuscular activity of the hand flexor muscles was observed. Hence, neural adaptations may only in part explain the present findings, and we cannot exclude the possible contribution from muscular adaptation to the training-induced increase in fatigue resistance. In support of this notion, musculoskeletal pain has previously been associated with impaired microcirculation in the painful muscles, reduced capillarization of muscle fibers, and lowered carbohydrate oxidation capacity [34–36]. Consequently, such local muscular factors may contribute to increasing anaerobic metabolism and thus influence fatigue development during various work tasks. In line with this notion, Andersen and coworkers reported that increased strength capacity during repetitive isometric shoulder contractions was paralleled by increased capillarization and myofiber hypertrophy following 10 weeks of strength training in women with trapezius myalgia [15]. In addition, heavy-resistance strength training is known to consistently induce a shift towards an increased proportion of fatigue-resistant fast-twitch (MHC IIA) myofibers along with a downregulation in the proportion of fatigable fast-twitch (MHC IIX) fibers [37, 38], in turn resulting in a more fatigue-resistant muscle fiber profile [39]. Even though no muscle biopsies were obtained in the present study, it is reasonable to assume that comparable muscular adaptations may have contributed to the improved fatigue resistance observed following strength training intervention in the present study.

The observed increase in handgrip strength (11%) following the period of strength training corresponds to previous reports in subjects with and without musculoskeletal pain in response to short-term strength training [16, 20]. Noticeable, the increase in maximal strength presently observed following 10 wks of strength training indicates that a higher absolute force output was needed at follow-up to avoid test completion (i.e., ≥50% peak force) in this group of workers. Hence, the increase in time to fatigue observed with strength training intervention not only reflects an improved relative fatigue resistance of the chronically painful muscles, but also strongly suggests that absolute fatigue resistance (i.e., time to contraction failure at the absolute load corresponding to 50% MVC measured at baseline) was improved as well. In contrast, a 16% decrease in maximal strength unexpectedly was observed following intervention based on ergonomic usual care, which similarly may have impacted (in this case negatively) fatigue test outcome at follow-up in this control group. The ergonomic training regime (exposure reduction) performed in this study is generally considered a useful treatment modality for the prevention of musculoskeletal disorders in slaughterhouse workers, and thus it seems unlikely that this intervention modality* per se* was the cause behind the observed decrease in maximal lower-arm strength. More likely, seasonal variation in pain symptoms and subjective health complaints may have influenced the results as the baseline questionnaire was administered in August and September and follow-up took place in December-January. In support of this potential scenario, Takala and coworkers reported decreased neck and shoulder pain from autumn and winter towards spring in female office workers [40], and Persson et al. showed that subjective health complaints were the highest in December to February in hospital workers [41]. In support of this notion, we have previously observed a significant worsening in work ability accompanied by a minor increase in work disability (although the latter did not reach statistical significance) with ergonomic intervention, which we ascribed to seasonal variations in pain symptoms [42]. Thus, the worsening in muscle strength following ergonomic usual care, compared with strength training, could be due to seasonal variation.

Chronic musculoskeletal pain is a multifactorial experience composed of a multitude of complex biopsychosocial interactions, and functional capacity assessments in these individuals are determined by biological, psychological, and social factors [17]. Fear avoidance (i.e., the belief that fast and forceful movements exacerbate pain) along with musculoskeletal pain itself is examples of psychological factors that can influence patients physical performance [17, 43], which in turn might inhibit efferent neural motor drive during a fatiguing trial and thus limit work capacity of painful muscles [16]. In support of this notion, Andersen et al. [16] observed a relatively greater increase in rate of force development (61%–108%) compared with maximal strength (20%–30%) following 10 weeks of strength training in women with trapezius myalgia. The authors speculated that a reduction in pain in concert with a change in fear avoidance contributed to these findings; however, pain-related beliefs were not quantified to support this hypothesis. In the present study we did an exploratory analysis on the interventional effect on fear avoidance beliefs, measured by a simple tailor-made single-item question. That analysis showed a group-by-time interaction for fear avoidance (*P* = 0.03; Figure 3). Specifically, fear avoidance was reduced more following strength training compared to ergonomic usual care. In addition, we estimated the associations (correlation coefficients) between specific variables (Table 3) and found that the pre- to postintervention change in fear avoidance was negatively correlated with the change in time to fatigue (Spearman's *r* = −0.40; *P* = 0.01) and peak extensor EMG during the fatigue test (−0.40; *P* = 0.017). However, the change in fear avoidance was unrelated to the change in handgrip strength (0.17; *P* = 0.31). Overall taken, these exploratory analyses showed that the change in fear avoidance following the strength training regimen was associated with the changes (gains) in both time to fatigue and hand extensor muscle activity during the fatigue test. Thus, it could be suggested that the training-induced change in fear avoidance may have contributed to the improved fatigue resistance (i.e., increased time to fatigue) of the chronically painful muscles possibly through reduced neural inhibition and/or increased voluntary motor drive. Since this was an exploratory analysis using a simple tailor-made single-item question to measure fear avoidance, the results should be interpreted with caution. In addition, the question on fear avoidance did not directly or discretely ask the subject about fear avoidance of work. However, the question still gives additional knowledge on pain rehabilitation among this occupational group, as slaughterhouse work tasks frequently encompass fast and forceful movements of the arm, shoulder, and hand. Future interventional studies on individuals with chronic pain should however apply more standardized questions on fear avoidance beliefs such as the Fear Avoidance Beliefs Questionnaire.

Importantly, the regime of strength training intervention appeared far more effective than ergonomic usual care in improving self-rated health. Self-rated health is a major independent predictor of objective health, morbidity, and mortality [44, 45] and symptoms such as chronic pain and fatigue are particular important constituents of self-rated health. Additionally, chronic pain is independently and significantly related to self-rated health [46] while impaired general health is associated with poor recovery from chronic pain [47]. Thus, it is possible that the observed decreases in chronic pain scoring observed following strength training was the main cause behind the observed improvement in self-rated health. Thus, specific strength training targeting chronically painful muscles not only seems to be a potent tool to reduce chronic musculoskeletal pain and to increase physical capacity, but also appears to provide an effective overall health promotion strategy.

### 4.1. Strengths and Limitations

The randomized controlled study design with concealed allocation and blinded clinical examiners protected against systematic bias. Additionally, similar levels of outcome expectations were observed at baseline to the two intervention protocols concerning the anticipated effectiveness on chronic pain relief (survey data not reported), which suggests that systematic placebo effects were unlikely to differentially have affected the two intervention groups. As a potential limitation of the study missing fatigue test data (participants not performing the fatigue test at baseline and follow-up) was observed in both intervention groups (strength training: *n* = 6 corresponding to 20%, ergonomic training: *n* = 2 corresponding to 7%). This inherently decreased the statistical power related to identifying significant between-group differences for this parameter. The fatigue test was performed as the last test in the test battery, and for logistic reasons not all participants had time to complete this test. This likely explains the larger amount of missing data in the fatigue test versus the MVC. However, due to the large effect size observed for the changes from baseline to follow-up in the strength training group versus ergonomic usual care, the observed between-group differences remained highly statistically significant. As another potential study limitation no muscle biopsies were obtained to examine the muscular adaptations that potentially might have contributed to the observed improvements in fatigue resistance in the strength training group. In addition, the results on fear avoidance should be interpreted with caution as we used a simple tailor-made single-item question to measure fear avoidance. Future interventional studies on individuals with chronic pain should apply more standardized questions on fear avoidance beliefs such as the Fear Avoidance Beliefs Questionnaire. Finally, the exclusion and inclusion criteria used in the present study confine the generalizability of our results to alone comprise individuals engaged in manual repetitive/vigorous work tasks with chronic pain symptoms in the arm, shoulder, and hand regions.

## 5. Conclusions

Specific strength training improves muscular fatigue resistance, elevates self-rated health, and reduces pain of chronically painful muscles of the hand/wrist region in slaughterhouse workers with chronic musculoskeletal pain and work disability. These exercise-induced adaptations were observed to have significant functional impact on fatigue resistance capacity during high-load upper limb work. As such, the present findings hold important implications for the maintenance and rehabilitation of physical health in individuals with chronic pain and work disability that are exposed to intense and repetitive manual work tasks.

## Figures and Tables

**Table 1:** Baseline characteristics of the two intervention groups.

	Strength training	Ergonomic training (usual care)
*n*	33	33
Number of men/women	25/8	26/7
Anthropometry		
Height, cm	174 (10)	177 (9)
Body mass, kg	83 (20)	86 (17)
Body mass index, kg·m^−2^	28 (6)	28 (5)
Age, year	48 (9)	43 (9)^*∗*^
Clinical		
Pain intensity of the hand/wrist (0–10)	3.9 (2.8)	3.7 (2.6)
Self-rated health (1–5)	3.0 (0.7)	2.9 (0.7)
Fear avoidance (1–4)	2.6 (0.4)	2.6 (0.4)
Handgrip MVC		
Strength (Newton)	375 (116)	372 (133)
Extensor EMG (mV)	182 (58)	181 (80)
Flexor EMG (mV)	226 (107)	226 (105)
Fatigue test		
Time to fatigue (sec)	24.2 (13)	22.9 (11)
Impulse (Ns)	6759 (2200)	6409 (3800)
Extensor peak EMG (mV)	193 (62)	189 (64)
Flexor peak EMG (mV)	282 (153)	283 (130)
Extensor mean EMG (mV)	148 (52)	146 (51)
Flexor mean EMG (mV)	208 (111)	211 (103)

EMG denotes “electromyography” and MVC denotes “maximal voluntary contraction.” Values are means (SD). ^*∗*^Difference between groups at baseline, *P* < 0.05.

**Table 2:** Interventional changes in strength and fatigue development.

	Difference from baseline to follow-up		Between group difference at follow-up		Group by time
	Strength training	Ergonomic training (usual care)	Strength versus ergonomic	*P* value	*P* value
Hand grip MVC					
Strength (Newton)	43 (5 to 81)	−61 (−96 to −26)	107 (69 to 144)	<0.0001	<0.0001
Extensor peak EMG (mV)	23 (−3 to 49)	−1 (−26 to 23)	25 (−2 to 52)	0.06	0.18
Flexor peak EMG (mV)	1 (−40 to 43)	−2 (−39 to 35)	3 (−39 to 45)	0.89	0.90
Fatigue test					
Time to fatigue (sec)	23.5 (14.6 to 32.5)	−7.0 (−96.9 to 82.7)	24.0 (14.6 to 33.4)	<0.0001	<0.001
Impulse (Ns)	6470 (4308 to 8633)	304 (−1883 to 2492)	6516 (4245 to 8787)	<0.0001	<0.001
Extensor peak EMG (mV)	47 (16 to 77)	−11 (−43 to 20)	62 (30 to 95)	<0.001	<0.01
Flexor peak EMG (mV)	−3 (−55 to 49)	−3 (−57 to 51)	−1 (−58 to 56)	0.98	0.99
Extensor mean EMG (mV)	26 (3 to 49)	−8 (−32 to 16)	36 (11 to 61)	<0.01	<0.05
Flexor mean EMG (mV)	−13 (−54 to 27)	2 (−40 to 44)	−18 (−62 to 26)	0.41	0.60
MPF extensor (Hz)	−7 (−13 to −2)	0 (−6 to 6)	−7 (−13 to −1)	<0.05	0.08
MPF flexor (Hz)	−5 (−13 to 3)	−4 (−12 to 5)	−2 (−11 to 7)	0.63	0.79

Changes in handgrip and fatigue test performance from baseline to 10-week follow-up. Associated changes in electromyography (EMG) are also illustrated. Differences of each group are illustrated on the left, and contrasts between the groups at follow-up in the middle. *P* values for the group by time interactions are shown on the right. MPF denotes “median power frequency.” Values are means (95% confidence interval).

**Table 3:** Correlation between variables.

	Correlation coefficient (Spearman's *r*)	
	Fear avoidance	Pain
Time to fatigue	−0.40^*∗*^	−0.29
Peak extensor EMG	−0.40^*∗*^	−0.24
Handgrip strength	0.17	0.13

Coefficient (Spearman's *r*) between pre- and postintervention changes in fear avoidance, pain intensity, time to fatigue, peak extensor EMG (during the fatigue test), and handgrip strength. ^*∗*^ *P* < 0.05.
